# Patient experiences of tissue donation and digital consent support in primary craniospinal tumour research

**DOI:** 10.1007/s00520-026-11017-x

**Published:** 2026-07-18

**Authors:** Gerard Mawhinney, Helen Higham, Simon Leedham, Olaf Ansorge

**Affiliations:** 1 Nuffield Department of Clinical Neurosciences, University of Oxford, Oxford, UK; 2 Nuffield Department of Medicine, University of Oxford, Oxford, UK

**Keywords:** Tissue donation, Informed consent, Craniospinal tumour, Digital consent, Rare cancer, Patient experience

## Abstract

**Purpose:**

Requests for tissue donation for research are often made at times of heightened vulnerability, particularly around diagnosis and surgery. This study explored patient experiences of tissue donation discussions, perspectives on consent, and the acceptability of digital decision support in primary craniospinal tumour research.

**Methods:**

A UK national online cross-sectional survey was conducted with 50 adults with a primary brain tumour or spinal sarcoma. The survey was developed with patient and public involvement; six patient contributors reviewed the initial questionnaire before launch. Descriptive statistics summarised closed responses, and open-text comments were grouped descriptively to contextualise quantitative findings. Reporting was informed by STROBE guidance.

**Results:**

Just over half of participants reported being invited to donate tissue for research (26/50, 52%). Respondents strongly preferred tissue donation to be discussed at or after a clinic appointment, and none selected the day of surgery as the preferred time. Among invited respondents, most reported that information was easy to understand (22/26, 85%), that they had an opportunity to ask questions (23/25, 92%), and that they had sufficient time to consider the decision (23/26, 88%). Sixteen of 26 invited respondents (62%) discussed the decision with family or friends; among invited respondents who had not done so, 7/10 (70%) would have liked the opportunity. Interest in a secure digital adjunct was high (46/49, 94%).

**Conclusion:**

Overall experience was generally positive, but the data identify specific, practical opportunities to strengthen consent support in rare craniospinal tumour pathways, including appropriate timing, clear and revisitable information, opportunities for question-asking, and resources that support family-inclusive decision-making.

**Supplementary Information:**

The online version contains supplementary material available at 10.1007/s00520-026-11017-x.

## Introduction

Primary tumours of the craniospinal axis are rare, heterogeneous malignancies associated with substantial morbidity and premature mortality. In the UK, brain and other central nervous system tumours, together with bone and soft tissue sarcomas, constitute a small proportion of all cancers, yet contribute disproportionately to illness burden and early death [1, 2]. Their rarity presents persistent challenges for diagnosis, evidence generation, and access to specialist services, in keeping with broader European efforts to improve outcomes in rare cancers [3, 4].

Requests for tissue donation for research sit within this context. Access to well-consented tumour tissue underpins translational cancer research, including genomic profiling, molecular stratification, and the development of targeted therapeutic approaches [5–8]. In primary brain tumours, progress remains constrained by biological complexity, treatment resistance, and limited access to representative biospecimens [9]. In primary spinal tumours, emerging clinical and research pathways increasingly rely on high-quality tissue linked to integrated clinical and molecular data [10].

Yet these conversations often occur around diagnosis and surgery, when patients may be overwhelmed, distressed, and less well placed to absorb complex information. Patient-centred communication and shared decision-making can support participation, understanding, and quality of care [11–15].

In biobanking contexts, concerns commonly relate to trust, governance, and clarity regarding how samples and associated data will be used, with additional vulnerabilities for people living with cancer [16–19]. Tissue-donation consent should therefore be understood as an ethically significant research decision that requires more than a procedural signature. Clear communication, appropriate timing, opportunities for questions, and time for reflection are central to ethically robust informed consent, particularly in rare tumour pathways where patients may be facing diagnosis and treatment decisions concurrently.

Evidence is growing for digital and dynamic consent approaches that improve transparency, revisitable information access, and participant control over time [20, 21]. However, little is known about how individuals with primary craniospinal tumours experience requests for tissue donation for research, how they perceive consent discussions, or whether digital tools might support more informed and patient-centred decision-making. This study therefore examined patient experiences of tissue-donation discussions, preferences regarding the timing and format of consent conversations, decision-support needs, and the acceptability of a secure digital adjunct in primary craniospinal tumour research.

## Methods

### Design and reporting

A national online cross-sectional survey was conducted. Reporting was informed by the Strengthening the Reporting of Observational Studies in Epidemiology (STROBE) statement for cross-sectional studies and the Consensus-Based Checklist for Reporting of Survey Studies (CROSS) [22, 23].

### Patient and public involvement

The survey was developed with patient and public involvement. Six patient contributors reviewed the initial questionnaire and provided feedback on the survey domains, wording, tone, clarity, and acceptability before launch. Their feedback informed minor changes to question wording and ordering. Survey participants were adults with primary craniospinal tumours; family members, carers, and clinicians were not eligible to complete the survey.

### Survey development

Survey content and framing were informed by a scoping review, patient and public engagement activities, and the wider PiCTuRE programme protocol [24–29]. PiCTuRE (Putting the Person in the PiCTuRE) is an exploratory sequential mixed-methods programme examining how people with primary craniospinal tumours experience precision medicine, tissue donation, and consent processes. This survey represented the quantitative survey phase of the wider programme. Prior work collectively highlighted uncertainty about risks and implications, trust and governance concerns, emotional burden at diagnosis, timing of recruitment, and feeling rushed. These insights shaped items on timing preferences, information clarity, opportunities to ask questions, family involvement, and preferences for ongoing, revisitable information via a digital adjunct.

### Participants and recruitment

Eligible participants were adults aged 18 years or older, living in the UK, with a primary tumour of the brain or spine, able to provide informed consent, and able to complete an English-language online survey. Recruitment was promoted through social media, UK brain tumour and sarcoma charities, and patient networks. A pragmatic sampling approach was adopted in view of the rarity of the target population and the exploratory aims of the study, consistent with sampling approaches described in applied health research [30]. The inclusion and exclusion criteria used to define eligibility for participation are summarised in Table 1.

**Table 1:** Inclusion and exclusion criteria

Inclusion criteria	Exclusion criteria
Aged 18 years or older	Under 18 years
Lived experience of a primary tumour of the brain or spine	No lived experience of a primary craniospinal tumour
Able to provide informed consent	Unable to provide informed consent
Able to understand English and complete the survey online	Unable to communicate effectively in English or complete the online survey

### Survey content

Survey items captured demographic characteristics and diagnosis group; invitation to donate tissue; emotional response to the request; preferred timing and format of discussions; perceived clarity of information; opportunities to ask questions; decision support, including discussion with family or friends; and acceptability and feature priorities for a secure NHS-grade digital information and consent adjunct.

### Data analysis

Closed items were summarised using descriptive statistics (counts and within-item percentages) [31]. Percentages were calculated using item-specific denominators because of item non-response and survey branching. Items relating to the experience of being asked about tissue donation were analysed among respondents who reported being invited to donate tissue for research. The follow-up item on whether respondents would have liked the opportunity to discuss the decision with family or friends was analysed among invited respondents who reported that they had not discussed the decision with family or friends. Digital readiness items were summarised among all respondents. Open-text responses were reviewed and grouped descriptively to contextualise the quantitative findings. Given the exploratory design and modest sample size, analyses were descriptive rather than inferential; no subgroup or association testing was undertaken.

### Ethics

This study was reviewed and approved by the Medical Sciences Interdivisional Research Ethics Committee (MS IDREC) (R79248/RE001), Medical Sciences Division, University of Oxford, UK. All participants provided informed consent electronically before completing the survey. The study was conducted in accordance with the Declaration of Helsinki.

## Results

### Participant characteristics

Fifty adults completed the survey. The sample included 34 respondents with spinal sarcoma (68%) and 16 with brain tumour diagnoses (32%). Most participants were female (62%). Ethnicity was White (70%), with representation from Asian, Black, Arab, and mixed ethnic groups. Age was broadly distributed; the largest age bands were 46–55 years (22%), 36–45 years (20%), and 66–75 years (18%). Time since diagnosis was derived from free-text responses; the median was 1.3 years, and approximately 71% of respondents were within three years of diagnosis. The demographic and clinical characteristics of the sample are summarised in Table 2.

**Table 2:** Participant characteristics (base *n* = 50 unless stated)

Characteristic	Category	*n* (%)
Gender at birth	Female	31 (62%)
	Male	19 (38%)
Diagnosis group	Spinal sarcoma	34 (68%)
	Brain tumour	16 (32%)
Ethnicity	White	35 (70%)
	Asian	5 (10%)
	Black	4 (8%)
	Arab	3 (6%)
	Mixed	3 (6%)
Age distribution	Largest age bands: 46–55 years, 36–45 years, 66–75 years	11 (22%), 10 (20%), 9 (18%)
Time since diagnosis (*n* = 49)	Median 1.3 years; within 3 years of diagnosis	35 (71%)

### Invitation to donate and preferred timing

Just over half of participants reported being invited to donate tissue for research (26/50, 52%); 17/50 (34%) reported not being invited, and 7/50 (14%) were unsure. When asked about the best time to introduce tissue donation, respondents answering this item preferred planned discussions at the clinic appointment (29/47, 62%) or after the appointment (13/47, 28%). Five of 47 respondents (11%) preferred written information before the appointment, and no respondents selected the day of surgery as the preferred time.

### Experience quality and decision support

Experience quality and decision-support findings are summarised in Table 3. Among respondents who reported being invited to donate tissue for research, 10/25 (40%) reported that being asked was stressful. Most invited respondents reported that the information was easy to understand (22/26, 85%), that they had an opportunity to ask questions (23/25, 92%), and that they had sufficient time to consider the decision (23/26, 88%).

**Table 3:** Consent discussion indicators and decision-support needs

Item	Population/denominator	Yes *n* (%)	No/other *n* (%)
Invitation to donate tissue	All respondents (*n* = 50)	26 (52%)	No: 17 (34%); unsure: 7 (14%)
Stressful to be asked	Invited respondents answering item (*n* = 25)	10 (40%)	15 (60%)
Information easy to understand	Invited respondents (*n* = 26)	22 (85%)	4 (15%)
Opportunity to ask questions	Invited respondents answering item (*n* = 25)	23 (92%)	2 (8%)
Sufficient time to consider	Invited respondents (*n* = 26)	23 (88%)	3 (12%)
Discussed with family/friends	Invited respondents (*n* = 26)	16 (62%)	10 (38%)
Would have liked to discuss	Invited respondents who had not discussed with family/friends (*n* = 10)	7 (70%)	3 (30%)
Interested in digital adjunct	All respondents answering item (*n* = 49)	46 (94%)	3 (6%)

Percentages are calculated using item-specific denominators. Items relating to the experience of being asked about tissue donation are analysed among respondents who reported being invited to donate tissue. The family/friend follow-up item is analysed among invited respondents who reported that they had not discussed the decision with family or friends

Overall experience ratings among invited respondents were positive (median 8/10; mean 7.85/10). Family and relational decision-support needs remained evident: 16/26 invited respondents (62%) had discussed the decision with family or friends, while 10/26 (38%) had not. Among invited respondents who had not discussed the decision with family or friends, 7/10 (70%) would have liked the opportunity to do so.

### Digital readiness and desired portal content

Digital engagement was high in this sample. Most respondents already used digital portals (48/50, 96%) and were willing to access consent information through a portal (48/50, 96%). Interest in a secure digital adjunct was high (46/49, 94%), while reported security concerns about an NHS-grade portal were low (4/50, 8%). Participants most frequently prioritised diagnosis-specific information (46/49, 94%), frequently asked questions (39/49, 80%), links to charities or support organisations (37/49, 76%), and information about active research projects (32/49, 65%). Open-text responses also suggested value in peer support links, mechanisms for asking questions, and optional updates on how donated tissue contributes to research.

**Fig. 1:**
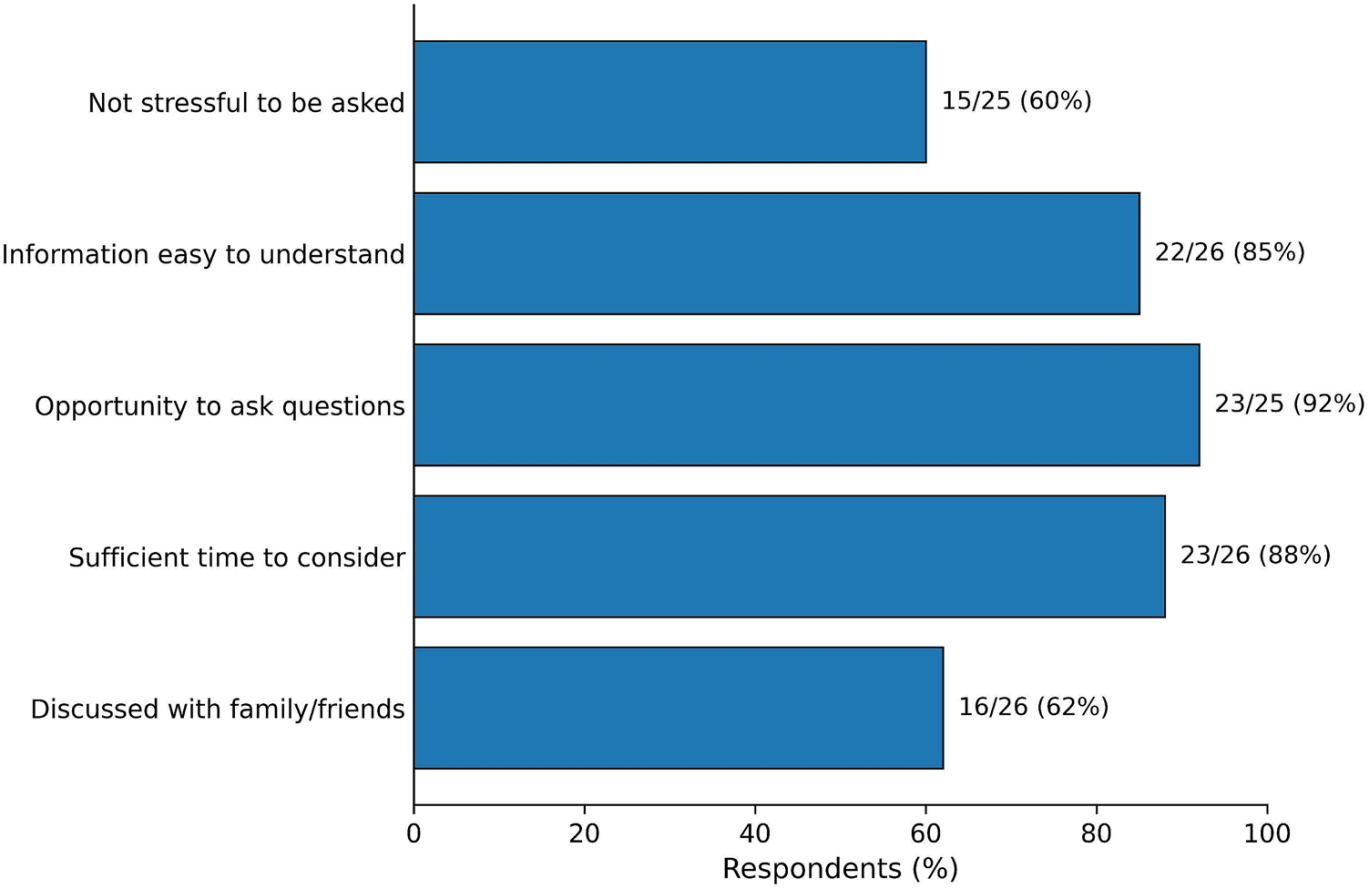
Consent-experience indicators among respondents invited to donate tissue for research

Figure 1 summarises consent-experience indicators among respondents who reported being invited to donate tissue for research. Most invited respondents reported understandable information, opportunities to ask questions, and sufficient time to consider the decision. The data also identify practical areas for improvement, including the emotional burden of being asked and opportunities for family or friend discussion.

Bars show the proportion and count of invited respondents reporting that being asked was not stressful, that information was easy to understand, that they had an opportunity to ask questions, that they had sufficient time to consider the decision, and that they discussed the decision with family or friends. Denominators vary because of item non-response.

**Fig. 2:**
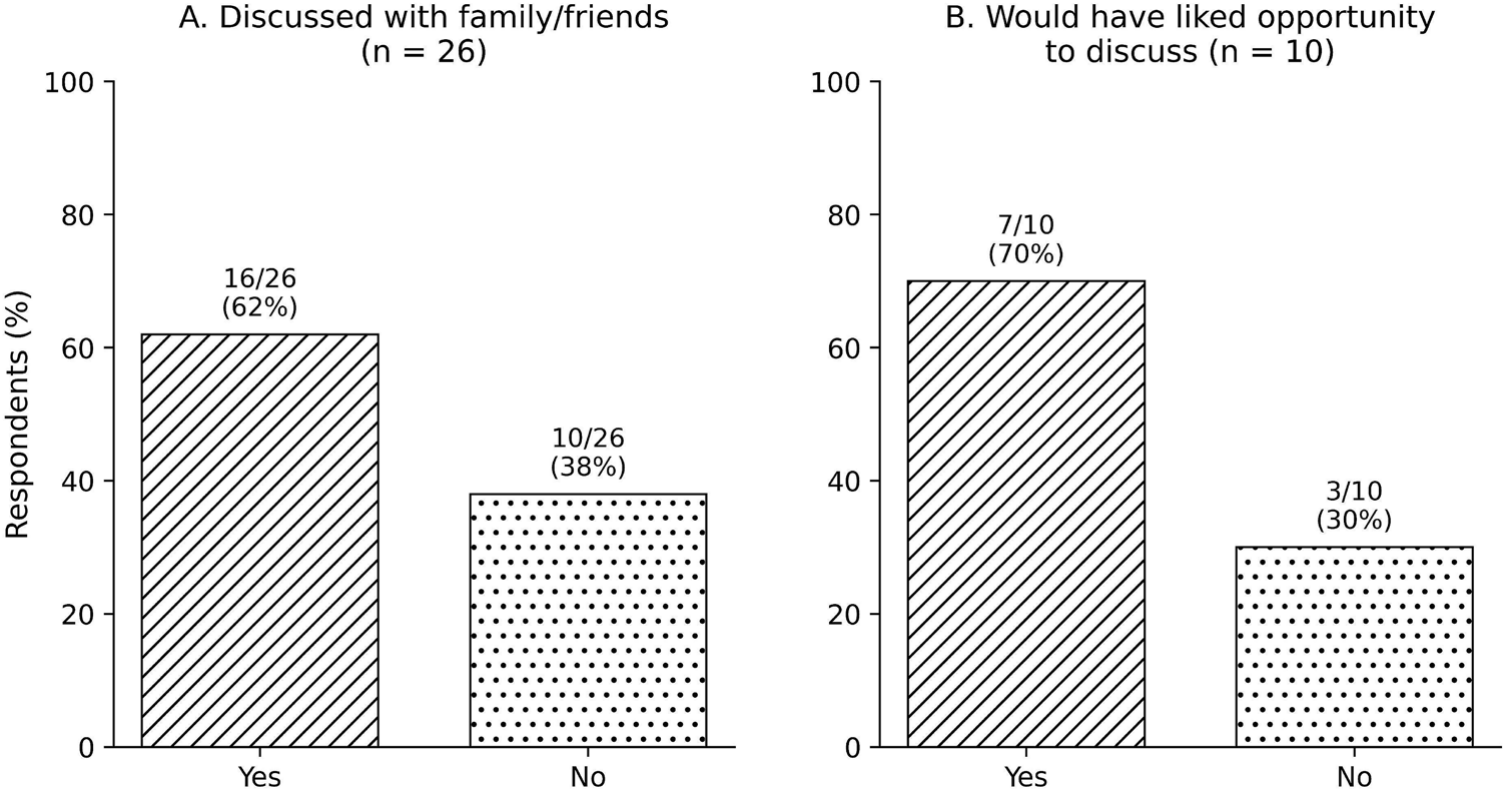
Family involvement and relational decision-support needs among respondents invited to donate tissue for research

Figure 2 further illustrates the relational dimension of tissue-donation decision-making. Among invited respondents, 10/26 (38%) had not discussed the decision with family or friends; of these, 7/10 (70%) reported that they would have liked the opportunity to do so.

Panel A shows whether invited respondents discussed the tissue-donation decision with family or friends. Panel B shows preferences for family or friend discussion among invited respondents who had not done so. Percentages use the number of respondents answering each item as the denominator.

## Discussion

In this national survey of adults with primary craniospinal tumours, participants generally supported tissue donation for research and reported broadly positive experiences when invited to donate. At the same time, the findings identify several modifiable aspects of consent support, including timing, emotional burden, family-inclusive decision support, revisitable information, and opportunities for questions. Tissue-donation consent should therefore be approached as an ethically significant research decision that requires clear communication, time for reflection, and careful attention to the clinical context in which the request is made. In this national survey of adults with primary craniospinal tumours, participants generally supported tissue donation for research and reported broadly positive experiences when invited to donate. At the same time, the findings identify several modifiable aspects of consent support, including timing, emotional burden, family-inclusive decision support, revisitable information, and opportunities for questions.

These findings are consistent with earlier work showing that the timing of consent influences perceived autonomy and burden, and that patients value opportunities to ask questions, deliberate, and make values-based choices [12–15, 32, 33]. They are also broadly in keeping with survey evidence from other cancer populations, including international studies reporting high receptiveness to biospecimen donation alongside the importance of trust, transparency, and preferences regarding consent and data sharing in shaping participation [16–19, 34, 35]. The findings therefore sit within the wider oncology research-consent and biobanking literature, while adding specific insight from a rare craniospinal tumour population.

In this study, no respondent selected the day of surgery as the preferred time for discussion. This finding is clinically relevant in rare craniospinal tumour pathways, where diagnosis, treatment planning, and operative decision-making may occur under considerable emotional strain. The data do not establish that day-of-surgery consent is routine in current practice; rather, they identify a patient preference that should inform future pathway design and evaluation. In rare tumour settings, where research tissue may be particularly valuable, consent processes should also avoid creating perceived pressure to donate and should make clear that declining will not affect care.

High reported acceptability of a secure digital adjunct suggests that revisitable information may offer a useful complement to clinician-led research consent discussions. In this context, digital support should not be regarded as a replacement for interpersonal consent conversations, but rather as a means of improving access to understandable information, enabling questions over time, and supporting shared and family-inclusive decision-making. These functions are especially relevant in rare tumour pathways, where patients may have limited prior knowledge, face considerable uncertainty, and encounter research participation decisions at clinically sensitive moments.

Family and social support also emerged as a targeted area for improvement. Although most invited respondents had discussed the decision with family or friends, a minority had not, and most of this subgroup would have liked the opportunity to do so. This aligns with biobanking literature suggesting that participation is often relational and shaped by trust, values, and the meaning attached to contributing to research [16–20]. Supportive resources that can be revisited and shared beyond the clinic encounter may therefore improve not only comprehension but also the experience of decision-making.

The manuscript has several limitations. The study was descriptive and based on a modest sample, albeit one that is typical of rare-tumour research. Denominators varied across items because of non-response and survey branching. Although individual-level data were reviewed to resolve skip-pattern denominators, the small sample size limited subgroup comparisons and association testing. Recruitment through charities and social media may have over-represented people who were already digitally engaged or motivated to contribute to research, and the English-language online format may have excluded people with different communication, language, or digital access needs. Combining participants with brain tumours and spinal sarcomas may have obscured diagnosis-specific differences in pathways and informational needs. These findings should therefore be interpreted as exploratory and hypothesis-generating.

Nevertheless, the study offers novel insight into tissue-donation discussions in a rare and clinically complex cancer population. By identifying specific priorities relating to timing, communication, family involvement, and revisitable information, this study offers actionable targets for improving consent support around research participation. These include introducing tissue donation before the day of surgery, providing clearer diagnosis-relevant information, enabling questions over time, and offering resources that patients can revisit and share with family or friends.

## Conclusion

People with primary craniospinal tumours identified actionable priorities for improving tissue-donation discussions, particularly in relation to timing, clarity of information, opportunities for questions, and relational decision support. Overall experience was generally positive, but the data identify specific, practical opportunities to strengthen consent support in rare craniospinal tumour pathways. Integrating these elements into clinical-led conversations and supportive digital resources may improve how research participation is discussed and experienced within rare craniospinal tumour pathways.

## Supplementary Information

Below is the link to the electronic supplementary material.

ESM 1 (PDF 19.4 KB)

ESM 2 (PDF 30.9 KB)

ESM 3 (PDF 143 KB)

ESM 4 (PDF 166 KB)

## Data Availability

No datasets were generated or analysed during the current study.
