# Supplementary figures and images for: Patient experiences of tissue donation and digital consent support in primary craniospinal tumour research
Source: Support Care Cancer. 2026 Jul 18;34(8):774. doi: 10.1007/s00520-026-11017-x (PMC13380566; doi:10.1007/s00520-026-11017-x)

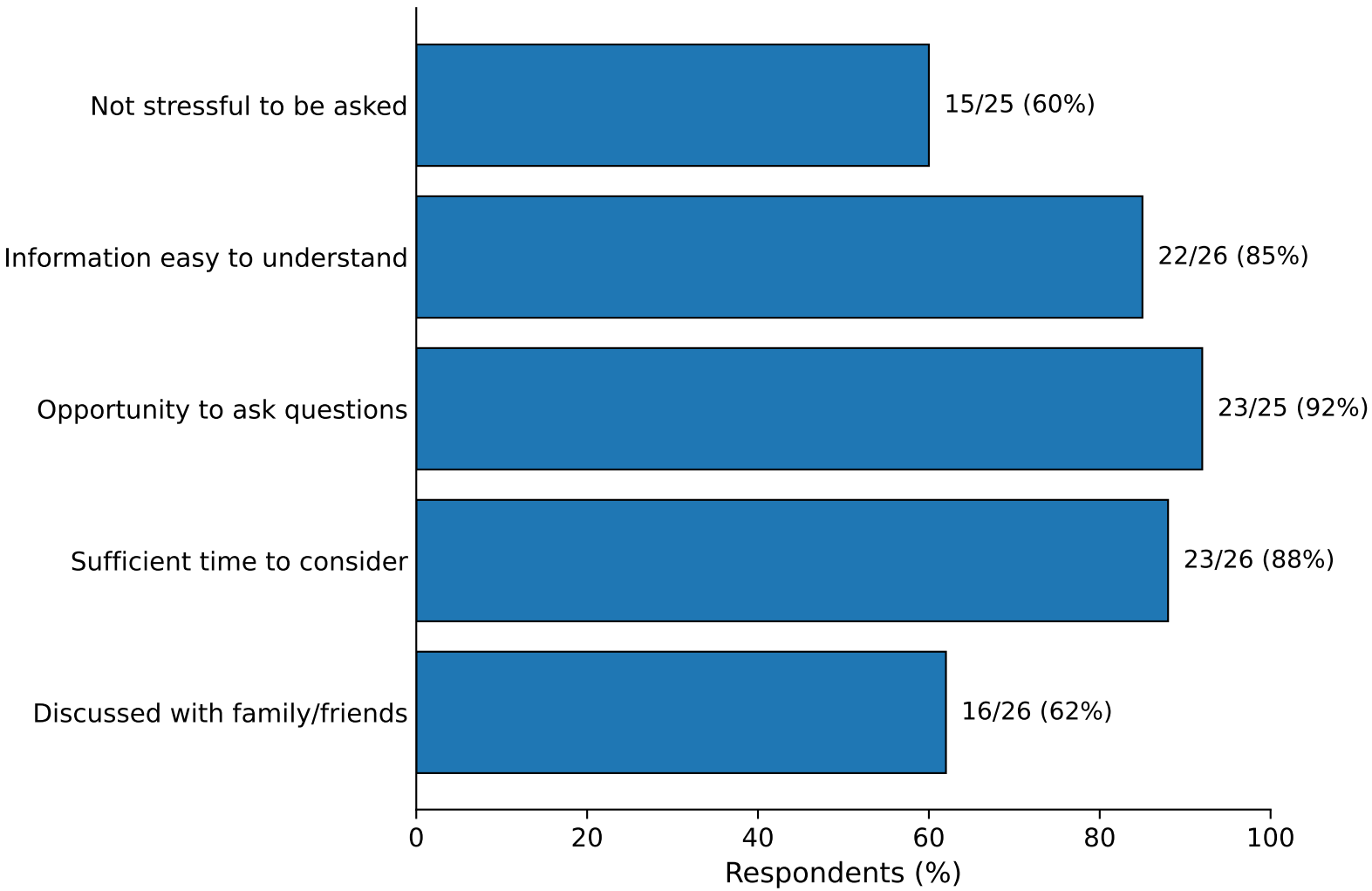

Supplement: Supplementary file 1 — (PDF 19.4 KB) [file 520_2026_11017_MOESM1_ESM.pdf]

A. Discussed with family/friends  
(n = 26)

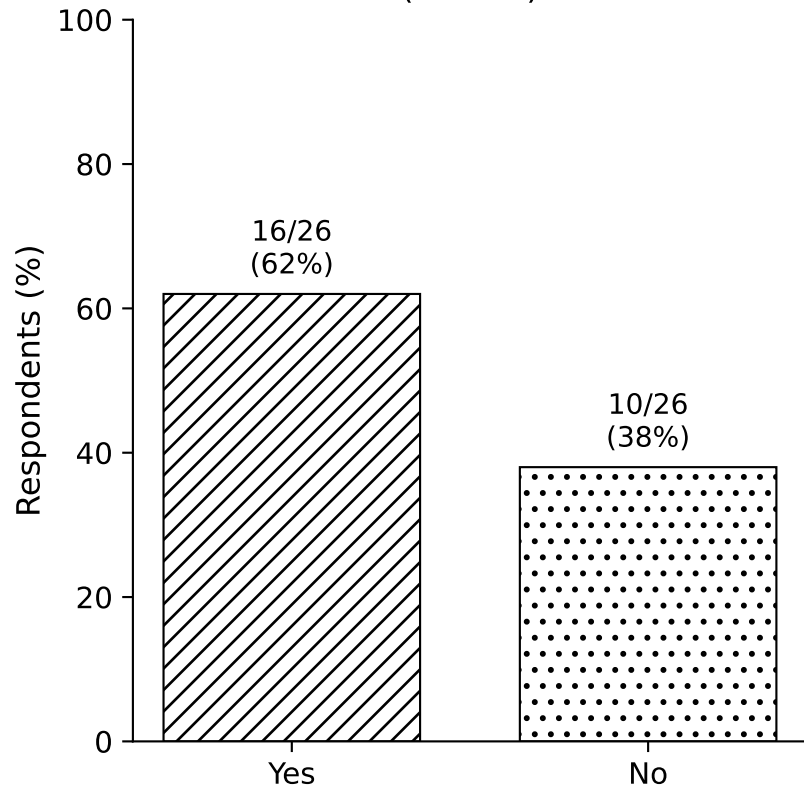

B. Would have liked opportunity  
to discuss (n = 10)

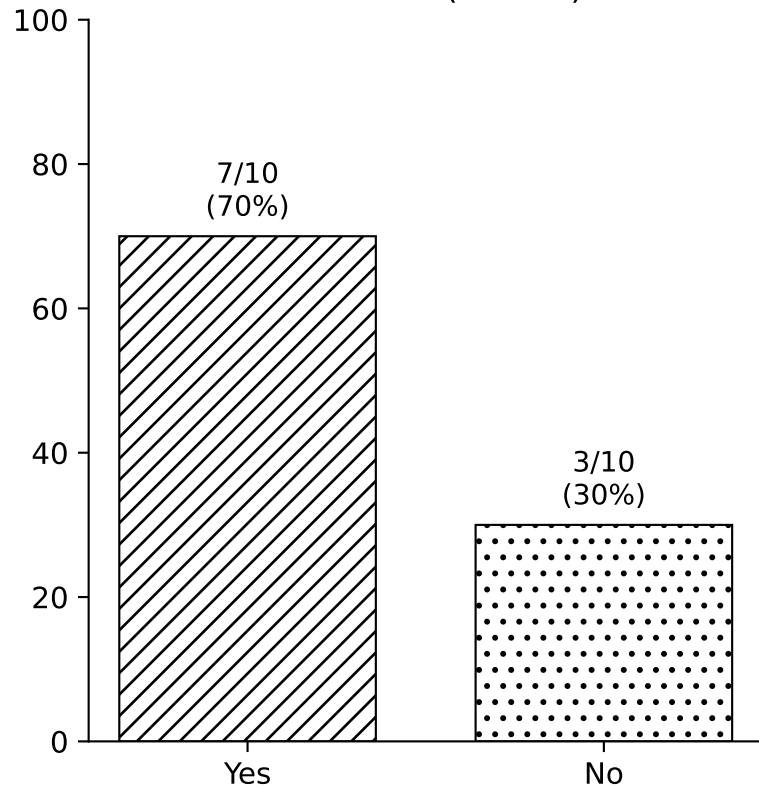

Supplement: Supplementary file 2 — (PDF 30.9 KB) [file 520_2026_11017_MOESM2_ESM.pdf]
