# Supplementary material for: Patient experiences of tissue donation and digital consent support in primary craniospinal tumour research
Source: Support Care Cancer. 2026 Jul 18;34(8):774. doi: 10.1007/s00520-026-11017-x (PMC13380566; doi:10.1007/s00520-026-11017-x)
Supplement: Supplementary file 3 — (PDF 143 KB) [file 520_2026_11017_MOESM3_ESM.pdf]

STROBE checklist for cross-sectional studies

Manuscript: Patient experiences of tissue donation and digital consent support in primary craniospinal tumour research

Item	Section	Recommendation	Page no(s)	Location in manuscript
1	Title and abstract	(a) Indicate the study design with a commonly used term in the title or abstract. (b) Provide an informative and balanced summary in the abstract.	1-2	Title and structured abstract revised to include co-design/piloting and balanced results.
2	Background/rationale	Explain the scientific background and rationale for the investigation.	2-4	Introduction paragraphs 1-4; consent framing revised.
3	Objectives	State specific objectives, including any prespecified hypotheses.	4	Final Introduction paragraph.
4	Study design	Present key elements of study design early in the paper.	4	Methods: Design and reporting.
5	Setting	Describe the setting, locations, and relevant dates, including recruitment and data collection.	6	Methods: Participants and recruitment; online UK recruitment via charities, social media and patient networks.
6	Participants	Give the eligibility criteria and sources and methods of selection of participants.	6-8	Methods: Participants and recruitment; Table 1; eligibility clarified as adults with primary craniospinal tumours.
7	Variables	Clearly define outcomes, exposures, predictors, potential confounders, and effect modifiers.	6	Methods: Survey content.
8	Data sources/measurement	For each variable of interest, give sources of data and details of methods of assessment.	6	Methods: Survey content and Supplementary File 1 survey instrument.
9	Bias	Describe any efforts to address potential sources of bias.	6-7, 16-17	Recruitment/design described in Methods; item-specific denominators and limitations discussed.
10	Study size	Explain how the study size was arrived at.	6	Methods: Participants and recruitment (pragmatic exploratory rare-tumour sample).
11	Quantitative variables	Explain how quantitative variables were handled in the analyses.	6-7	Methods: Data analysis; item-specific denominators and invited-respondent analyses described.
12	Statistical methods	(a) Describe all statistical methods. (b) Describe methods for subgroup analyses/interactions. (c) Explain how missing data were addressed. (d) If applicable, describe analytical methods taking account of sampling strategy.	6-7, 10-13	Descriptive statistics described in Methods; item-specific denominators reported in Results, Table 3 and Figures 1-2; no subgroup analyses undertaken.
13	Participants	(a) Report numbers of individuals at each stage. (b) Give reasons for non-participation. (c) Consider use of a flow diagram.	8	Results opening paragraph; total completing sample reported. Flow diagram not used.
14	Descriptive data	(a) Give characteristics of study participants. (b) Indicate number with missing data for each variable.	8-11	Table 2 and Table 3; item-specific denominators reported.
15	Outcome data	Report numbers of outcome events or summary measures.	9-13	Results section, Table 3 and Figures 1-2.
16	Main results	(a) Give unadjusted estimates and, if applicable, confounder-adjusted estimates. (b) Report category boundaries when	9-13	Descriptive unadjusted counts and percentages

		continuous variables were categorised. (c) If relevant, consider translating estimates into absolute risk.		reported; no adjusted analyses applicable.
17	Other analyses	Report other analyses done, such as subgroup and sensitivity analyses.	6-7, 16-17	No subgroup or sensitivity analyses were undertaken; this is stated in Methods and Limitations.
18	Key results	Summarise key results with reference to study objectives.	14-17	Discussion opening paragraphs summarise key results in relation to study objectives.
19	Limitations	Discuss limitations of the study, taking into account sources of potential bias or imprecision.	16-17	Discussion limitations paragraph; includes sample size, item non-response/branching, digital self-selection, online English-language recruitment and generalisability.
20	Interpretation	Give a cautious overall interpretation considering objectives, limitations, multiplicity of analyses, and evidence from similar studies.	14-17	Discussion provides cautious interpretation with reference to objectives, limitations and wider oncology biobanking/research-consent literature.
21	Generalisability	Discuss the generalisability of the study results.	16-17	Discussion limitations and concluding interpretation paragraphs.
22	Funding	Give the source of funding and the role of the funders.	18-19	Funding statement and role of funder reported in Declarations.
