# Supplementary material for: Patient experiences of tissue donation and digital consent support in primary craniospinal tumour research
Source: Support Care Cancer. 2026 Jul 18;34(8):774. doi: 10.1007/s00520-026-11017-x (PMC13380566; doi:10.1007/s00520-026-11017-x)
Supplement: Supplementary file 4 — (PDF 166 KB) [file 520_2026_11017_MOESM4_ESM.pdf]

# Supplementary File 1. Blank survey instrument

Manuscript: Patient experiences of tissue donation and digital consent support in primary craniospinal tumour research

This supplementary file reproduces the survey items and response formats used in the online survey. No respondent-level data or contact details are included.

**1. Please confirm that you are 18 years of age or over.**

- I certify that I am 18 years of age or over

**2. If you have read the participant information and agree to participate with the understanding that the data may be used for research, please indicate whether you agree to take part.**

- Yes, I agree to take part

**3. Age**

- 18-24
- 25-35
- 36-45
- 46-55
- 56-65
- 66-75
- 76-85
- 86 or over

**4. What was your gender at birth?**

- Female
- Male
- Prefer not to say
- Other

**5. Which best describes your ethnicity?**

- White
- Asian
- Black
- Arab
- Mixed race
- Other
- Prefer not to say

**6. What is/was your diagnosis?**

- Brain tumour
- Spinal sarcoma

**7. What was the name of your tumour type? For example, glioma, chordoma, chondrosarcoma, or other.**

*Response format: free text.*

**8. How long have you had your diagnosis?**

*Response format: free text.*

**9. As part of your treatment journey, were you invited to donate tissue for research?**

- Yes
- No
- Not sure

**10. Did you find it stressful to be asked about taking part in research when faced with the possible diagnosis of a brain or spinal tumour?**

- Yes
- No

**11. If you found it stressful to be asked about donating tissue for research, please tell us why.**

*Response format: free text.*

**12. In your experience, when would have been the best time to introduce the topic of donating tissue for research?**

- In the letter inviting you to a consultation with a specialist
- At the time of your appointment
- After your appointment
- On the day of surgery

**13. When asked to participate in research, did you find the information easy to understand?**

- Yes
- No

**14. How was the information delivered?**

- Printed
- Electronic
- Verbal
- Other

**15. Did you have the opportunity to ask any questions?**

- Yes
- No

**16. Who consented you to take part in research?**

- Treating clinician (hospital)
- Research nurse
- Specialist nurse
- Other

**17. Where did the consent take place?**

- Clinic room with the treating clinician present
- In a different clinic room
- Waiting room
- Hospital ward
- Other

**18. Did you have sufficient time to consider your decision to participate or not?**

- Yes
- No

**19. Prior to agreeing to take part in research, did you have the opportunity to discuss your decision with family or friends?**

- Yes
- No

**20. If no, would you have liked the opportunity to discuss your decision with family or friends?**

- Yes
- No

**21. On a scale of 1-10, where 1 is the worst and 10 is the best, how would you rate your experience of donating tissue for research?**

- 1
- 2
- 3
- 4
- 5
- 6
- 7
- 8
- 9

- 10

**22. Do you have experience of using digital/online portals to access your personal information, such as online banking or healthcare applications?**

- Yes
- No

**23. Would you be happy to use a digital/online portal such as the NHS app to access information regarding your decision to donate tissue for research?**

- Yes
- No

**24. The PiCTuRE project will introduce the concept of digital dynamic consent. This means consent will be obtained through a secure digital platform and can be revisited over time. Would you be interested in this approach?**

- Yes
- No

**25. The portal will also contain additional resources. Which of the below would be of interest to you? Choose all that apply.**

- Active research projects
- Completed research projects
- Frequently asked questions section
- Information related to your primary tumour diagnosis
- Links to tumour charities
- Other

**26. If other, please tell us what additional information you would find useful.**

*Response format: free text.*

**27. The portal would be hosted on an NHS-grade secure online platform, complying with UK data protection and GDPR requirements. Would you have any concerns about using this platform?**

- Yes
- No

**28. If yes, please tell us your concerns.**

*Response format: free text.*

**29. If no, please tell us why.**

*Response format: free text.*

**30. We kindly ask a few participants to volunteer for an additional interview. Would you be willing to be contacted by the researcher?**

- Yes
- No

**31. Optional follow-up contact detail for interview volunteers.**

*Response format: contact detail field. This field is not reproduced and no contact details are included in this supplementary file.*
